# Successful transplantation of porcine liver grafts following 48-hour normothermic preservation

**DOI:** 10.1371/journal.pone.0188494

**Published:** 2017-11-27

**Authors:** Thomas Vogel, Jens G. Brockmann, David Pigott, Desley A. H. Neil, Anand S. Rathnasamy Muthusamy, Constantin C. Coussios, Peter J. Friend

**Affiliations:** 1 Nuffield Department of Surgical Sciences, University of Oxford, Oxford, United Kingdom; 2 Nuffield Department of Anaesthesia, University of Oxford, Oxford, United Kingdom; 3 Department of Cellular Pathology, Queen Elizabeth Hospital Birmingham, Birmingham, United Kingdom; 4 Institute of Biomedical Engineering, University of Oxford, Oxford, United Kingdom; Istituto Mediterraneo per i Trapianti e Terapie ad Alta Specializzazione, ITALY

## Abstract

Current cold storage organ preservation technique fails to preserve marginal donor grafts sufficiently. Evidence from large animal experiments suggests superiority of normothermic machine preservation of liver allografts. Long-term organ preservation using normothermic perfusion might not only allow organ viability assessment before transplantation, but also provide the means for further organ modifications under physiologic conditions. Previous research has shown that porcine livers can be transplanted successfully after normothermic preservation of 20 hours. In the present study we investigate whether similar methodology is capable of further extending the safe limit to 48 hours. In this study, livers from White Landrace pigs were preserved by normothermic, oxygenated sanguineous perfusion. After a 48-hour period of preservation, livers were transplanted into recipient pigs and followed for 5 days. Outcome parameters measured included markers of synthetic and metabolic liver function as well as hepatocellular injury and blood gas analysis during perfusion and follow-up. Histological assessment of morphological liver integrity was performed. All livers showed sustained bile production and metabolic activity throughout the preservation period. Low levels of hepatocellular damage were found. Following transplantation all liver grafts revealed excellent graft function and death-censored graft survival was 100%. Porcine livers were transplanted successfully following 48 hours normothermic machine preservation.

## Introduction

Increasing the safe preservation time in liver transplantation is desirable for a number of reasons. Prolonged preservation would greatly benefit the logistics of liver transplantation, enabling enough time to assess the viability of a donor organ more objectively before committing the patient to surgery. Beyond that, it would allow time for therapeutic interventions that might even improve the quality and viability of the organ.

Conventional static cold storage has changed little since the introduction of the University of Wisconsin solution in the early 1990s [1]. There are theoretical limits to the duration of preservation using static cold storage, based upon depletion of ATP, accumulation of metabolites (that provide the substrate for the ischemia-reperfusion cascade) and loss of cellular membrane functions.

The use of hypothermic machine perfusion may be effective in abrogating some of these problems, and indeed is showing considerable promise in early clinical trials [2]. Machine perfusion is likely to be effective in removing cellular metabolites and thereby reducing ischaemia-reperfusion injury at the time of implantation. Also the provision of oxygen, even at cold temperature, is known to be effective in improving organ viability [3]. However, even with the benefit of machine perfusion and oxygen delivery, cold preservation is still likely to compromise the subsequent function of the organ in a time-dependent manner.

Normothermic machine perfusion (NMP) appears to allow much longer periods of successful preservation than have been achieved using cold storage. We have previously demonstrated successful transplantation of porcine livers after 20 hours of normothermic preservation [4]. Notably, in this study, there appeared to be no deleterious effects of a prior period of 40 minutes of warm ischemia (a model of donation after circulatory death).

In this study, using similar methodology, we have successfully tested normothermic machine preservation in the context of a 48-hour period of organ preservation, using healthy porcine livers (a model of the ‘ideal’ DBD donor organ).

## Materials and methods

### Animals

Fifteen white Landrace pigs (30–40 kg body weight) were used for this study. Animals were treated in accordance with the Animal (Scientific Procedures) Protection Act 1986 of the United Kingdom. All experiments were covered by Project Licenses PPL 30/2258 and PPL 30/2750 granted by the Home Office of the UK. The animals were fasted for 12 hours prior to surgery with free access to water. Sedation with ketamin/midazolam was followed by endotracheal intubation and mechanical ventilation. Anaesthesia was maintained with inhalational isoflurane. Catheters were placed in the internal jugular vein (donor/recipient) and carotid artery (recipient only). Animals were killed according to schedule 1 of the Animal (Scientific Procedures) Protection Act 1986 with sodium-pentobarbitone at the end of experiments or if defined humane endpoints were met. Surgery was performed by specialist board certified transplant surgeons (PJF, JGB, TV).

### Donor operation

The standard technique used to retrieve liver grafts was employed. The steps of the operation have been described previously [5]. In brief: following laparotomy a cholecystectomy was performed and the liver was dissected until connected only by its vascular attachments. The common bile duct was ligated at its distal end and divided. IV heparin was administered, a 20-Fr cannula (Medtronic, Watford, UK) was placed into the distal aorta and cold perfusion commenced after aortic cross clamping (3 l, Soltran, Baxter). Following hepatectomy, livers remained cold during back-table preparation before connection to the circuit. During back-table preparation the diaphragmatic remnant was oversewn to ensure haemostasis during perfusion and cannulae were inserted into the suprahepatic inferior vena cava (IVC, 28Fr, Medtronic), the hepatic artery (10Fr, Medtronic) and the portal vein (20Fr, Medtronic). The bile duct was cannulated with a catheter of appropriate size (usually 6-8Fr) and the infrahepatic IVC was closed by a running suture. Back table preparation and cannulation time was recorded and adds to the preservation time.

### Perfusion device

**Fig 1:**
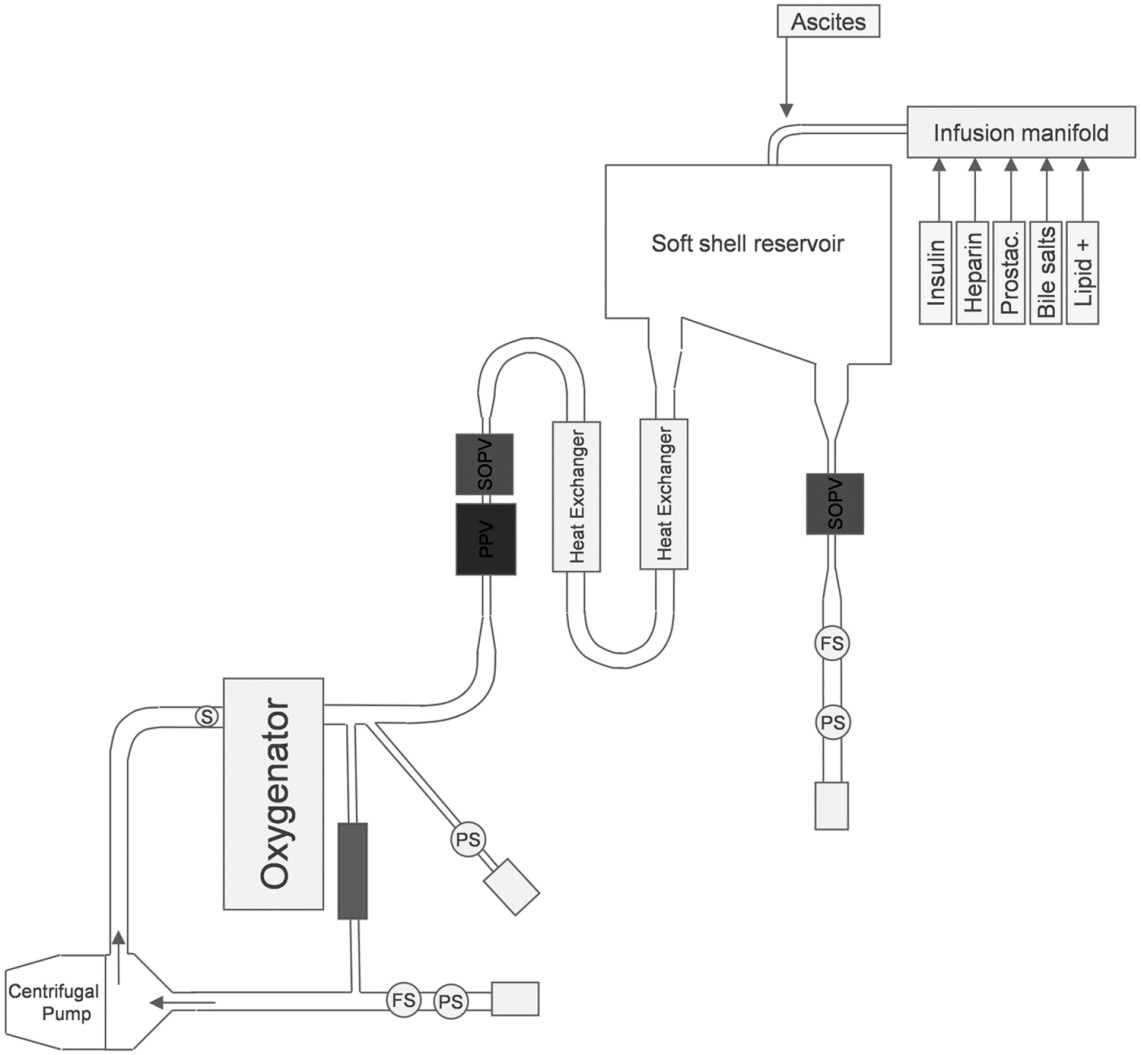
Diagram of perfusion circuit. Key: SOPV: Shut-off pinch valve, PPV: Proportional pinch valve, Shunt S.: Terumo Shunt Sensor, PS: Pressure Sensor, S: Sampling port, FS: Flow sensor. (Illustration not to scale).

In this study an automated perfusion apparatus was used. Basic tubing components were sourced from cardiopulmonary bypass suppliers. The principal components were: a centrifugal pump drive (Medtronic), hollow fibre membrane oxygenator (D905 EOS, Sorin, Italy) a soft-shell reservoir (Capiox 1500, Terumo) and medical grade silicone tubing (Raumedic AG, Germany) (Fig 1). The perfusion circuit was made up of an arterial limb being fed directly from the centrifugal pump (high pressure) and a portal limb, being fed passively from the venous reservoir by gravity (low pressure). Venous blood was drained via the IVC cannula and returned to the centrifugal pump, and then to the heater-oxygenator. All pressures (portal, hepatic artery and IVC) were measured using single use in-line pressure sensors (Pendo TECH, US). Perfusate flows within the portal vein, hepatic artery and IVC were monitored constantly by ultrasonic flowmeters. Oxygenation of the perfusate was controlled using a closed-loop system comprising an in-line blood gas analyser and software-controlled gas valves. The device was managed by a central processing unit integrating pump speed, valve resistances, perfusate flows and pressures in each limb of the circuit, temperature and perfusate oxygenation. Data relating to all parameters were continuously stored for later evaluation.

### Perfusate

The circuit was primed with 1.5 litres of pig blood and anticoagulated with heparin. At the start of perfusion the initial acidosis was corrected with sodium bicarbonate but no pH correction was made thereafter. Further bolus additions during priming comprised calcium gluconate and cefuroxime. Antibiotic prophylaxis was renewed after 24 hours.

### Liver perfusion

Before connection of the liver to the perfusion circuit, the preservation solution was flushed using 1 l of crystalloid solution at room temperature. This also allowed complete exclusion of air from the liver and cannulae. The liver then was placed on a sling in a sterile perfusion chamber. A drain at the most dependent part of the liver bowl was connected to the reservoir to recirculate any free fluid that collected (using a roller-pump). During preservation normal physiological flows and pressures were maintained in the hepatic artery, portal vein and IVC by controlling pump speed and gate clamp resistance of the reservoir inflow. The relative proportions of oxygen and air flow to the oxygenator were adjusted to maintain blood gases within physiological ranges (pO2: 10–20 kPa; PCO2: 3–8 kPa).

Prostacyclin (Flolan, GlaxoSmithKline, UK) at 8μg/hour, taurocholic acid (New Zealand Pharmaceuticals, NZ) at 140 mg/hour and heparin at 830 IU/hour were infused using constant flow syringe pumps at a flow rate of 1ml/hour. Nutrition was provided by an infusion of essential amino acids (Nutriflex plus, B.Braun AG, Germany) at 15ml/hour. Nutrition consisted of 96 g amino acids and 300 g anhydrous glucose in 1 litre solution. No lipid was given. Insulin was added to the perfusate depending on measured perfusate glucose levels. Bile production was measured hourly and perfusate samples collected.

### Recipient operation

All animals underwent the same implantation procedure. After midline laparotomy the liver was freed from its attachments. The structures of the hepatoduodenal ligament were isolated. The common bile duct and the hepatic artery were divided close to the liver. The portal vein, as well as the suprahepatic and infrahepatic inferior vena cava were isolated and the retrocaval space was prepared. Before removal of the native liver, the spleen was removed and a passive veno-venous shunt was placed between the splenic vein and external jugular vein. The portal vein, suprahepatic and infrahepatic inferior vena cava were clamped and hepatectomy was performed. In parallel, the liver graft was taken off the machine perfusion device and cannulae removed. For this period livers were flushed with ice-cold preservation solution. Following suprahepatic & infrahepatic IVC and portal anastomoses, the liver was reperfused through the portal vein before anastomosing the hepatic artery. Following transplantation the abdomen was closed. For surveillance and administration of IV drugs a catheter was placed into the internal jugular vein and tunnelled to exit at the neck of the animal. At the end of the operation, animals were extubated when breathing spontaneously and returned to their pens with free access to food and water. During the follow—up period pain was relieved by regular administration of opioid (morphine) and nonsteroidal anti-inflammatory medication (Carprofen, Rimadyl^®^, Pfizer, UK). Postoperative cyclosporine and steroids were given for immunosuppression. Welfare assessment of animals postoperatively was provided by round-the-clock surveillance for 36 hours, at least until first food intake and 6–8 hourly visits thereafter. A distress score sheet was used for the entire follow up period. Experiments were terminated at five days of follow-up or at humane endpoints. Animals were sacrificed after sedation by i.v. administration of pentobarbitone.

### Biochemistry

Perfusate (arterial) blood gases were monitored using an in-line blood gas analyser and recorded continuously. Venous blood gases obtained at the beginning of perfusion and on each hour during perfusion were analysed using a bench blood gas analyser. Blood glucose and bile production were also recorded every hour. The perfusate was sampled every 2 hours for the first 8 hours of the reperfusion phase and every 4 hours thereafter for biochemical liver function tests and full blood count. Following liver transplantation blood was drawn from the recipient pig every hour for the first 4 hours, thereafter at hours 6, 12 and 18 on the first day. From day 2 onwards blood samples were taken once daily.

For biochemistry, blood was immediately centrifuged and plasma was stored at -80°C until analysed.

### Histopathology

Biopsies were taken at donor hepatectomy and at the end of preservation. After termination of the experiment an autopsy was performed; the liver was sectioned and multiple random samples were obtained. At each time point, samples were fixed in formalin and snap frozen in liquid nitrogen and stored at -80°C until analysis.

At least 5 sections of samples stained with haematoxylin-eosin for morphological analysis were examined. The sections were scored using a semi-objective scale as previously published [6]. The sections were scored for necrosis, architectural destruction, apoptosis, sinusoidal congestion, sinusoidal dilatation, and hepatocellular vacuolization.

### Statistical analysis

All values are expressed as the mean ± standard deviation (SD) or numbers (%), as appropriate. Univariate comparisons of baseline factors were performed using Student's t or Mann-Whitney U tests for continuous variables and Fisher’s exact tests for categorical variables. Two-sided p-values of less than 0.05 were considered significant. Statistical analysis was performed using IBM SPSS Statistics (Version 22.0. Armonk, NY: IBM Corp.).

## Results

In total, five sets of experiments were performed (blood donor, liver donor, transplantation). During the third perfusion an interruption of the power supply to the perfusion device occurred. The liver was not perfused for a prolonged time and remained warm until restart of the machine. After restart of the device substantial changes in perfusion parameters were noted; this experiment was excluded from further analysis.

First cold ischemia times were (cold perfusion until start of normothermic reperfusion) were 76 minutes ± 19 minutes. This time encompassed final set-up of the perfusion apparatus as well as backtable preparation and cannulation of the liver graft. After normothermic preservation grafts were removed from the perfusion apparatus, cannulae removed and post-perfusion parameters assessed (e.g. biopsy, weight, and volume). Mean second cold ischemia time was 26 minutes (SD 12 minutes, range 15–38 minutes). Mean time for anastomosis was 41 minutes. (SD 5 minutes, range 36–47 minutes).

### Survival

#### Animal survival

**Fig 2:**
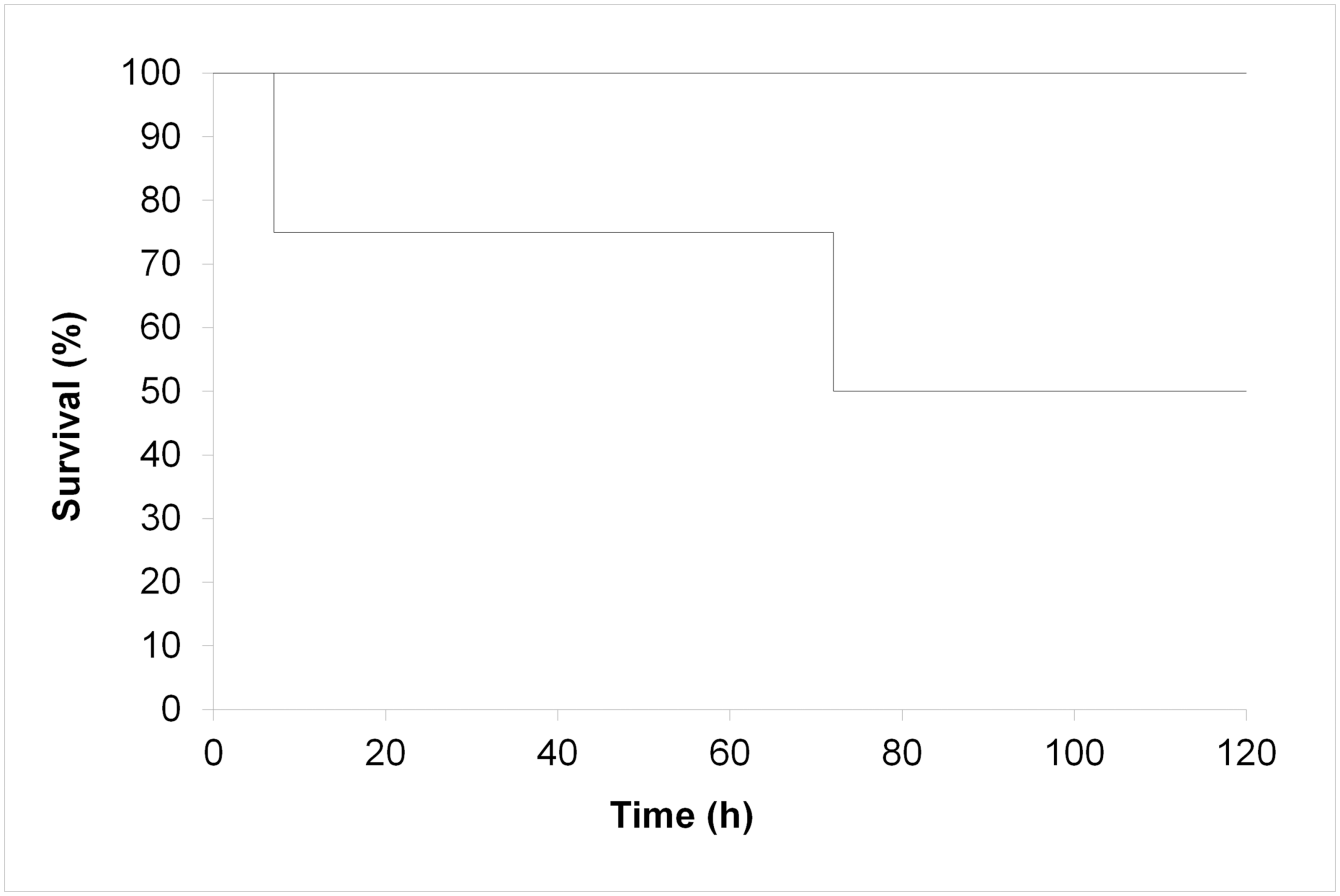
Animal survival and graft survival. Animal survival and graft survival censored for death with functioning graft.

Survival of the animals following orthotopic liver transplantation was 50% (Fig 2). One animal died from technical complications on day 1 (haemothorax due to bleeding from suprahepatic IVC anastomosis). Liver function parameters at time of death revealed good liver function. One pig developed severe distension of the abdomen on the third postoperative day. Relaparotomy showed obstructive ileus caused by torsion of the small bowel; the pig was euthanized. Macroscopic, microscopic and biochemical analysis of this liver also revealed good liver function and viable histological appearances up to the point of termination.

#### Graft survival

Death censored graft survival was 100% for 5 days of recipient follow up. All livers showed good and stable function until termination of the experiments. None of these experiments was terminated as a result of poor liver function.

### Perfusion parameters

All livers showed macroscopic normal appearance during perfusion and before transplantation. Liver weight before and after perfusion was not significantly different. Mean weight after hepatectomy and following preservation was 1226 g ± 143 g and 1464 g ± 94 g, respectively (p = 0.41). Liver volume after perfusion was slightly increased, though without reaching level of significance (liver volume before and after perfusion: 1189 ml ± 83 ml and 1450 ml ± 140 ml, respectively, p = 0.16).

#### Pressures / Flows

**Fig 3:**
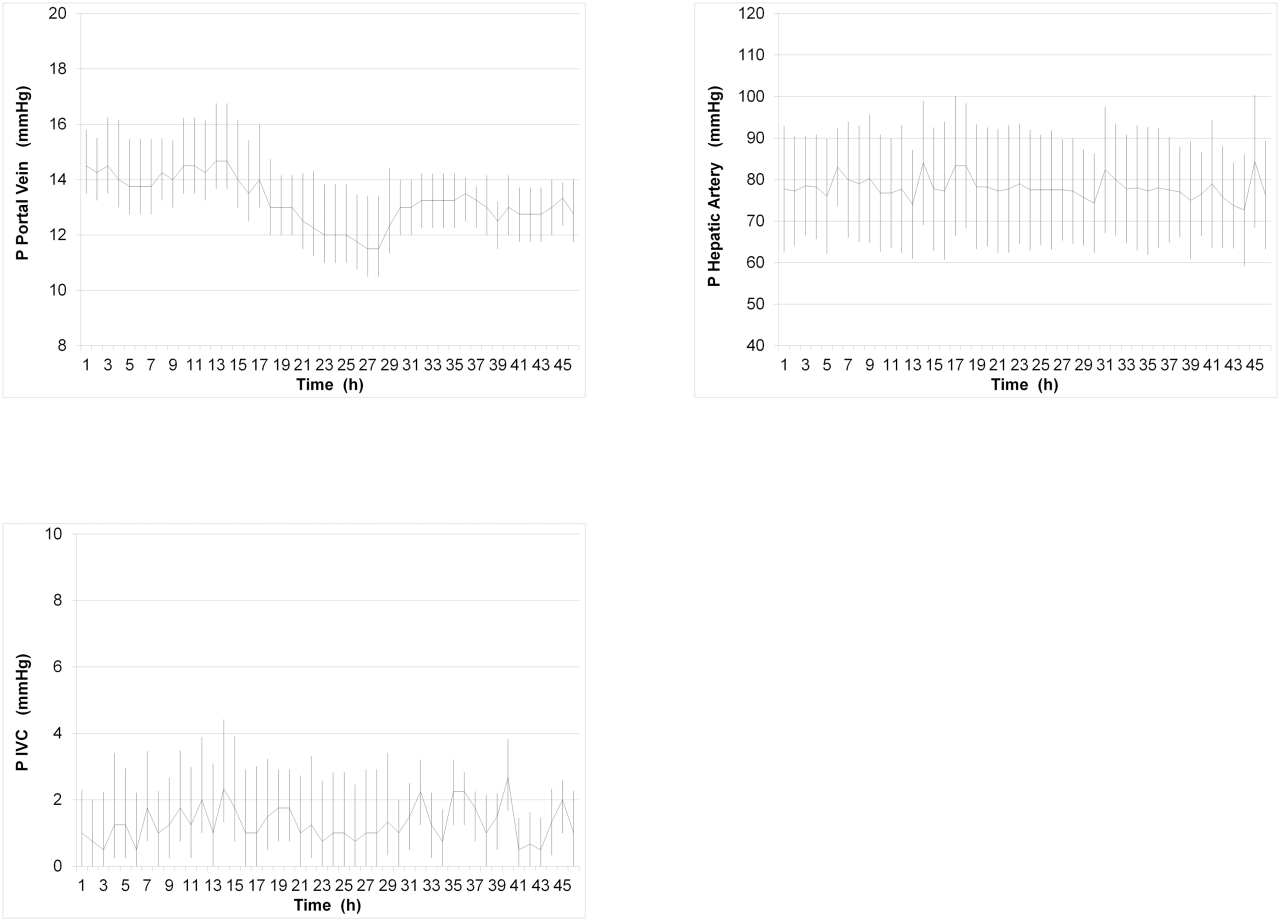
Mean pressures during 46 hours of normothermic perfusion. Control was achieved by regulation of pump speed and inflow to portal venous blood reservoir to keep values within physiologic range. Portal pressure was based on the principle of autoregulation. (A) Portal pressure ± SD, (B) Arterial pressure ± SD, (C) IVC pressure ± SD. All pressures were measured in-line showing little variation between livers. (n = 4).

**Fig 4:**
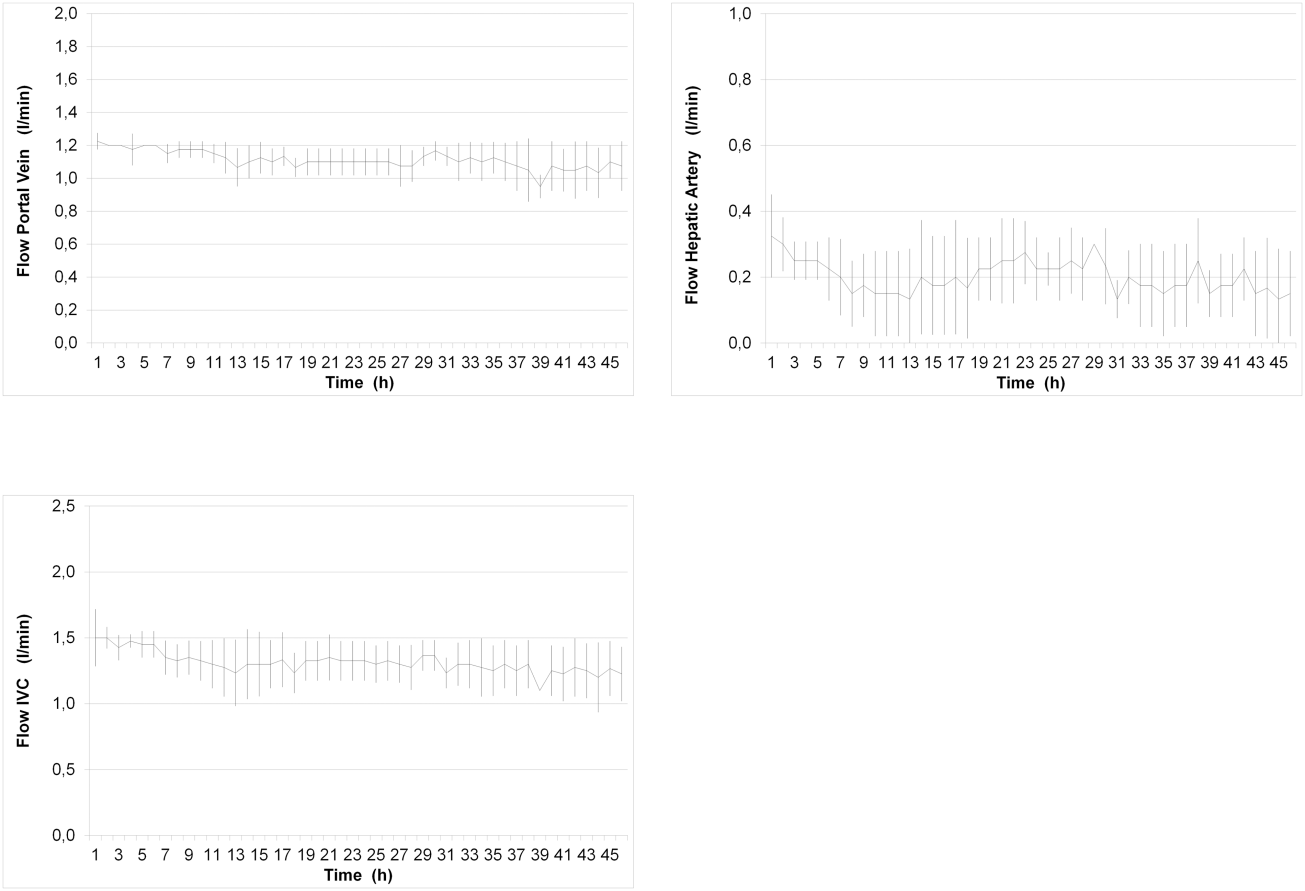
Perfusion flows during 46 hours of normothermic perfusion. (A) portal flow ± SD, (B) hepatic artery flow ± SD, (C) IVC flow ± SD. Perfusate flows were measured in-line using ultrasonic flow probes.

Arterial and IVC pressures were maintained within physiologic ranges. Mean portal vein, hepatic artery and IVC pressures were 13.2 mmHg ± 1.4 mmHg, 78.0 mmHg ± 13.8 mmHg and 1.3 mmHg ± 1.0 mmHg, respectively (Fig 3A–3C). The mean portal blood flow was 1.1 l/min ± 0.1 l/min throughout the entire normothermic perfusion with little variation between livers (Fig 4A). Mean arterial and total perfusion blood flows were 0.2 l/min ± 0.1 l/min and 1.3 l/min ± 0.2 l/min, respectively (Fig 4B and 4C).

#### Bile production

**Fig 5:**
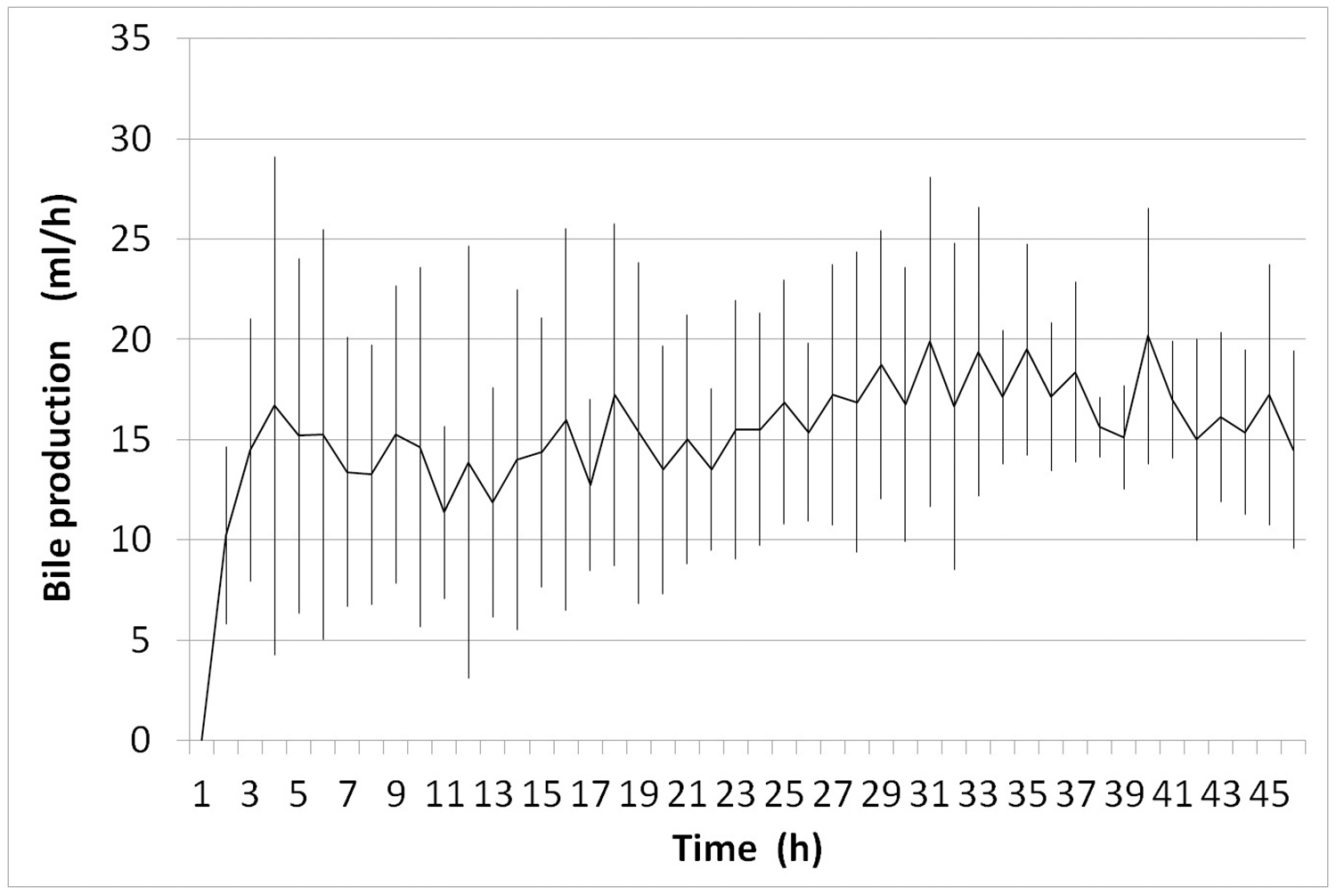
Mean bile secretion during normothermic organ preservation. During normothermic perfusion all livers produced clear, viscous bile. Secretion started within two hours of perfusion and was sustained until the end of the preservation period. (n = 4).

All livers showed bile production throughout the period of normothermic perfusion. The average bile production per hour was 15.7 ± 6.3 ml/h and remained above 9 ml/h until the end of the preservation period (Fig 5). Mean cumulative bile production was 366.9 ml for the first 24 hours of perfusion and 341.3 ml on day 2 (SD, ± 111.9 and ± 167.2, resp.).

#### Acid-base homeostasis

**Fig 6:**
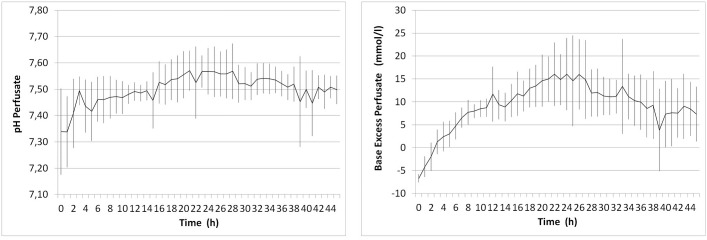
pH and base excess during normothermic organ preservation. Acid—base homeostasis was sustained until the end of organ preservation solely dependent on autoregulation of the perfused liver.

pH was adjusted to physiological levels prior to connection of the liver and all livers thereafter maintained pH levels which were within the physiological range, or progressively alkalotic, without further addition of bicarbonate. Mean pH was 7.41 ± 0.13 at hour 2 of perfusion and 7.50 ± 0.05 at hour 46 (Fig 6A). These data were mirrored by sustained homeostasis of bicarbonate and base excess in perfusate (Fig 6B).

#### Cellular injury

**Fig 7:**
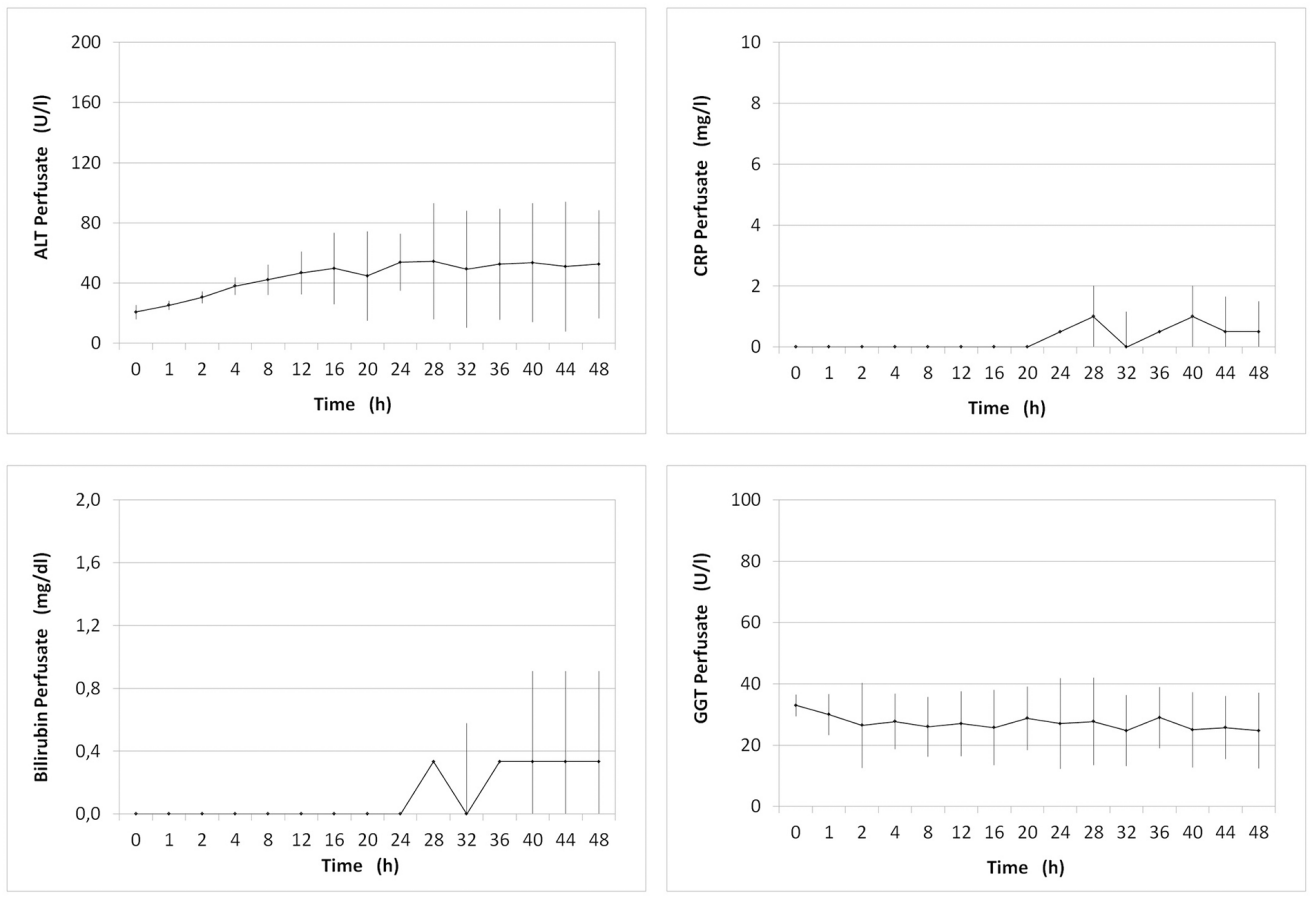
Markers of cellular injury. ALT, CRP, Bilirubin and GGT measured in perfusate during normothermic machine perfusion ± SD.

During normothermic perfusion markers for hepatocellular injury remained low. The mean ALT levels at 20 hours of perfusion were 44.8 ± 18.9 U/l and 52.5 ± 38.1 U/l at the end of the preservation period (Fig 7A). The rise in LDH mirrored that of ALT. Levels of CRP, bilirubin and γ-GT in perfusate also remained within physiological range during normothermic perfusion until the end of the preservation period (Fig 7B–7D).

### Post-transplant data

**Fig 8:**
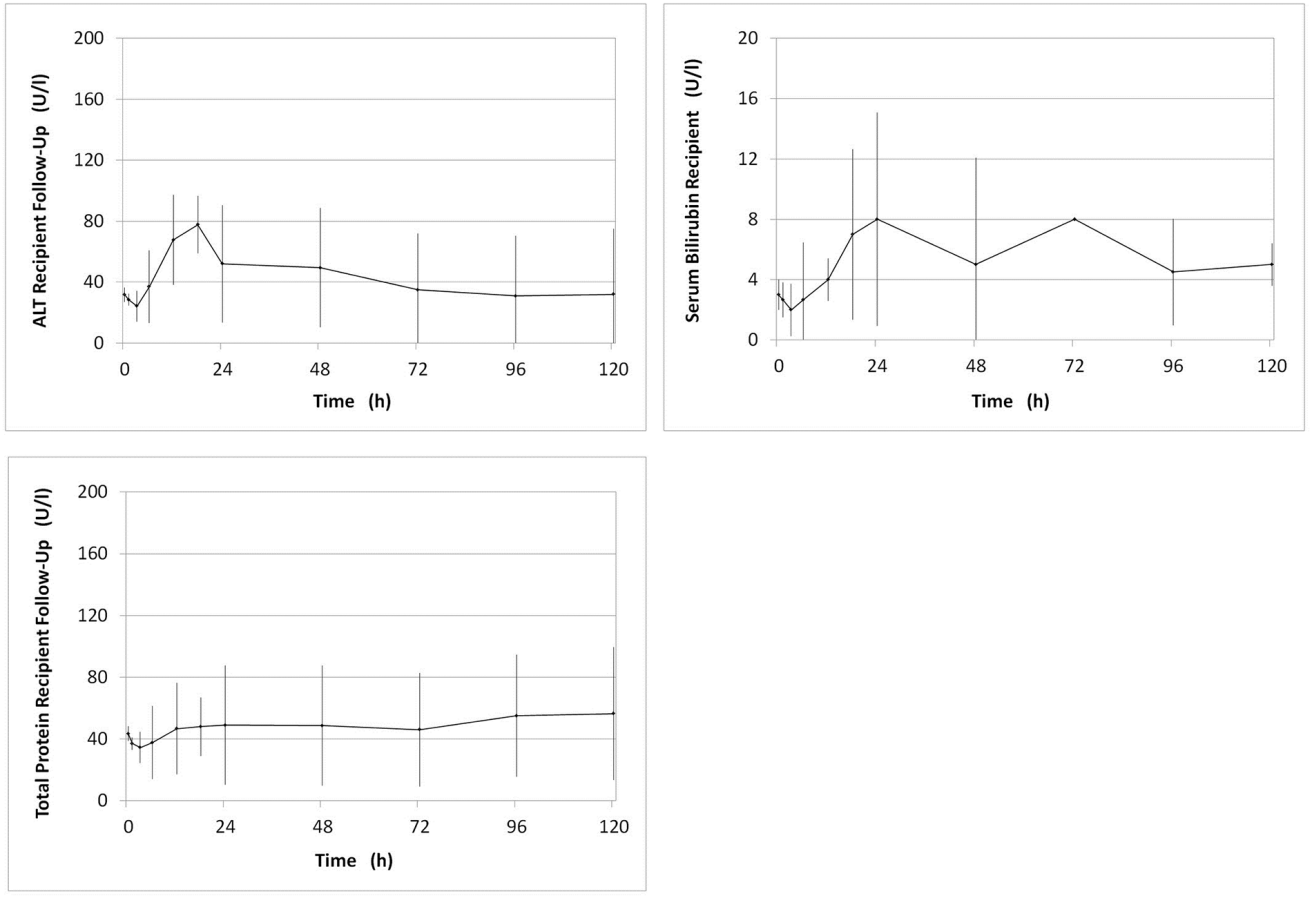
Post-transplant markers of cellular injury. (A) ALT (U/l), (B) bilirubin (mg/dl)*, (C) total protein (mg/dl) measured in serum during 5 days of follow-up after orthotropic liver transplantation ± SD. *In one pig autopsy revealed an anastomotic leak from the common bile duct. Serum levels of bilirubin were increased to above 20 mg/dl. Bilirubin data from this pig were excluded.

All livers showed prompt and sustained function following orthotopic liver transplantation. ALT serum levels remained low during 5 days of follow-up. On day five, mean ALT was 31.0 U/l ± 1.4 U/l. (Fig 8A) Hepatic synthetic function was also sustained after liver transplantation as could be seen by physiological protein levels during follow-up (Fig 8C).

On autopsy one pig was found to have anastomotic bile duct leakage with extravasation of bile within the abdomen. Systemic bilirubin levels in this animal (which survived clinically unperturbed) rose to above 20 mg/dl. In the graph shown the serum bilirubin data from this pig are excluded (Fig 8B).

**Fig 9:**
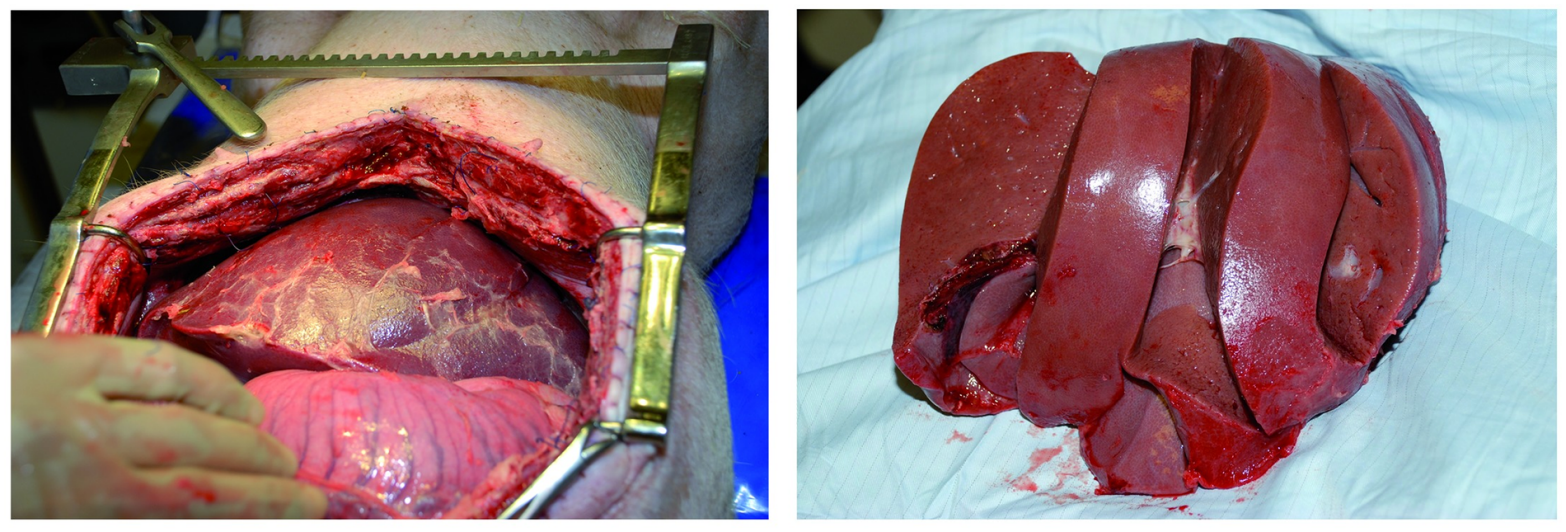
Liver at the end of perfusion and follow-up. (A) Post-transplant appearance of a liver after normothermic machine preservation for 48 hours; (B) porcine liver at autopsy following 48 hour normothermic preservation and five days of recipient follow-up.

After five days of follow-up animals were euthanized and an autopsy performed. The livers had healthy macroscopic appearances and texture. (Fig 9B)

### Histology

**Fig 10:**
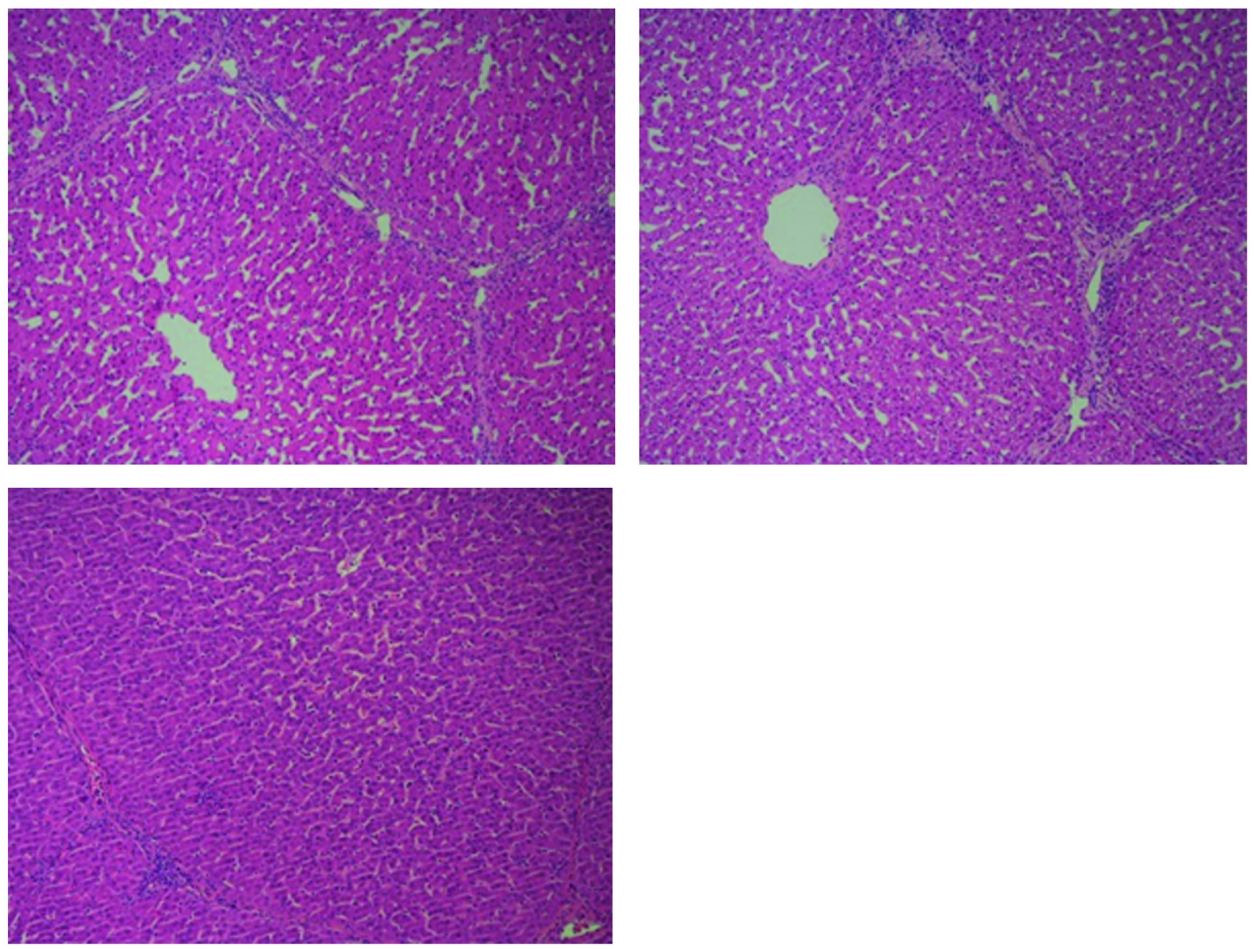
Histological appearance of liver parenchyma. (A) Biopsy at baseline (following donor hepatectomy), (B) Biopsy taken after 48 hours of organ preservation by normothermic machine perfusion, (C) post-mortem biopsy after five days of recipient follow-up.

In all post-perfusion samples histology revealed well-preserved structure of liver tissue (Fig 10A–10C). Discrete portal oedema was present with no signs of necrosis or apoptosis following 48 hours of organ preservation by normothermic machine perfusion. In addition, erythrocyte infiltration could be seen around portal veins. These infiltrates were not present in post-transplant autopsy results of animals surviving to 5 days.

Following transplantation some livers developed histological features of acute rejection, despite standard immunosuppression. At 5 days of follow-up livers revealed in most areas normal hepatocyte morphology with only mild or very mild focal coagulative necrosis (Fig 10C). The animal which died on day one due to bleeding complications showed signs of portal oedema and erythrocyte infiltration that were similar to those in the post-perfusion biopsies. No liver showed signs of steatosis, cholestasis or cholangitis.

## Discussion

Static cold storage is the most commonly used preservation method for all organs. The principles underlying cold preservation are slowing of metabolism (by cooling) and reduction of cell swelling due to the ionic composition of preservation solutions [7].

Preventing the depletion of cellular energy charge has the potential to improve graft quality substantially and is the main objective of normothermic, oxygenated preservation methods. In contrast to cold preservation, the purpose of normothermic preservation is to maintain cellular metabolism by replicating the physiological environment [5,8].

We have demonstrated in an early set of experiments that normothermic perfusion is capable of maintaining porcine livers in viable condition for durations of up to 72 hours [5]. In these experiments we have used ex-vivo machine perfusion as surrogate for transplantation and did not prove whether sustained markers of viability following long term preservation correlate with actual graft and patient survival. To date the maximum preservation times described in the literature for porcine liver transplantation studies range between 12 hours for cold preserved organs and up to 24 hours for machine perfused organs [9]. Bakker et al studied long term preservation in a porcine model of orthotopic and heterotopic liver transplantation. Survival in the extended preservation experiments was dismal. In orthotopic liver transplantation all animals died the day of the operation after 48 hr and 72 hr graft preservation. Another study published by Manner et al described no animal survival following 18 hours of cold preservation of healthy livers in a pig liver transplant model [10]. Steininger et al. conclude from a similar study that ′cold storage for 24 h is an extreme in pig liver preservation and is not compatible with animal survival′ [11]. Based on these data and in accordance with the regulations of the Animals (Scientific Procedures) Act of the United Kingdom we have not used a comparison group of livers undergoing 48-hours of cold storage preservation.

On the basis of the good results achieved with manually controlled perfusions we have developed a normothermic perfusion device with automated control of perfusion pressures, perfusate oxygenation and temperature. The system is pressure controlled for the hepatic artery and IVC; automated pinch valve control and pump speed algorithms provide stable perfusion pressures. This was reflected by stable perfusion flows during the entire preservation period of 48 hours. Moreover, resistance, as calculated by pressure over flow, remained unchanged. This is in contrast to what was described for hypothermic machine perfusion preservation [12]. In this study by van der Plaats et al. a significant decrease of portal and arterial flows were described in a pressure controlled setting of hypothermic machine perfusion of healthy porcine livers. The authors speculate that this increase in resistance might result from oedema formation during hypothermic perfusion.

Stable haemodynamic parameters provided the means for the fast recovery of liver function after reperfusion. Metabolic and synthetic liver function was sustained over the entire period of machine perfusion and transplantation outcome revealed excellent graft survival. Transaminase levels after transplantation revealed excellent preservation of liver grafts under normothermic conditions. Guarrera et al have performed liver transplantation experiments in pigs using hypothermic machine perfusion preservation [13]. After only 12 hours of either cold storage preservation or hypothermic machine perfusion preservation ALT levels in recipient animals were elevated above 2000 U/l. In contrast, transaminase release in the perfusate under normothermic machine perfusion for 48 hours and subsequent transplantation has not exceeded 130 U/l; mean levels were below 80 U/l at any time. Functional and synthetic parameters were stable beyond 40 hours, suggesting that even longer preservation times might be feasible. The impact of the length of preservation times under hypothermic conditions was already shown by Belzer et al in a pioneering paper [14]. Four out of five animals survived transplantation for 7 days after 8–10 hours preservation, but only two out of 12 survived beyond 12 h when the preservation period was extended to 24 hours.

The results described in this paper, albeit in a very small series, provide evidence that normothermic machine perfusion is capable of allowing very prolonged periods of liver preservation. There are real potential benefits in being able to preserve a donor organ for periods of 48 hours. If this can be achieved without detriment to the subsequent function and survival of the organ, then liver transplantation could become a semi-elective procedure. The logistics of operating departments, intensive care units and other aspects of the hospital infrastructure would be transformed by this level of flexibility. It would provide much more time for the retrieval, transport and assessment of the organ. Normothermic machine perfusion provides insight on viability and quality of the perfused liver; however, more research and experience is needed to accurately assess the different parameters advocated for viability testing in their clinical relevance. At the moment none of these data are robust enough to decide whether to accept or discard an organ [15,16]. Contrary to cold preservation technique (either static or perfused) normothermic preservation offers the opportunity to repair organs under physiological conditions. Several emerging novel techniques have been suggested for graft modification and improvement during the organ preservation period [17–19]. Most of these are strongly time-dependent and a means to extend preservation times might allow these techniques to transfer from the experimental to the clinical setting.

Normothermic machine perfusion of liver grafts has been advanced to clinical application [20]. The technical challenges of providing a normothermic perfusion device, capable of supporting the liver for periods of two days or more, are considerable. Many of the components in the circuit described in these experiments are manufactured for use in cardiopulmonary bypass circuits. These circuits are, typically, designed to be used for up to several hours but not for several days. However, comparable components are available for use in extracorporeal membrane oxygenation circuits, and these are designed to function for many days. Constant technical improvements will lead to superior results [21].

Clearly these results must be assessed in the context of what is a small study involving ideal pig organs. Future studies must investigate the extent to which these results are transferable from the experimental laboratory to the situation of sub-optimal human livers. However, in general, it is notable that the pig liver is a very rigorous model of organ preservation, with maximum successful preservation times (using conventional methods of cold storage) that are substantially shorter than those regularly achieved in clinical practice. There are grounds for optimism, therefore, that comparable benefits will be seen in the clinical environment.

Future studies will also need to investigate whether or not extending normothermic preservation time leads to progressive graft dysfunction; this is not been investigated in this or previous studies. If a stable physiological milieu can be maintained for the liver, it is possible that there is, in fact, little relationship between the duration of preservation and graft injury. If this were the case, then periods of preservation even longer than 48 hours would be a realistic objective.
